# Oral pathologic conditions and impaired cytokine response in patients with previous cerebral abscess or cervical necrotizing soft tissue infection

**DOI:** 10.3389/fcimb.2025.1547826

**Published:** 2025-05-30

**Authors:** Frederik Viktor Bang Jespersen, Signe Undall-Behrend Hansen, Sisse Rye Ostrowski, Claus Henrik Nielsen, Lars Haukali Omland, Simon Storgård Jensen, Merete Markvart

**Affiliations:** ^1^ Department of Odontology, University of Copenhagen, Copenhagen, Denmark; ^2^ Department of Oral & Maxillofacial Surgery, Copenhagen University Hospital, Copenhagen, Denmark; ^3^ Department of Clinical Immunology, Copenhagen University Hospital, Copenhagen, Denmark; ^4^ Department of Clinical Medicine, University of Copenhagen, Copenhagen, Denmark; ^5^ Institute for Inflammation Research, Department of Rheumatology and Spine Disease, Copenhagen University Hospital, Copenhagen, Denmark; ^6^ Department of Infectious Diseases, Copenhagen University Hospital, Copenhagen, Denmark

**Keywords:** cerebral abscess, necrotizing soft tissue infection, oral pathology, TruCulture, stimulated immune response

## Abstract

**Background:**

Microorganisms involved in cerebral abscess (CA) and cervical necrotizing soft tissue infection (NSTI) are frequently commensals of the oral cavity. Yet, the reasons for their ability to cause severe opportunistic infections remains unknown. The purpose of this study was to evaluate the dental characteristics and examine the stimulated immune response in patients with a history of CA or cervical NSTI.

**Methods:**

This observational study includes a clinical dental examination, and the collection of blood samples from 18 previous CA patients and four previous cervical NSTI patients. Whole blood cells were stimulated with LPS, R848, Poly I:C and anti-CD3/-CD28 beads in TruCulture tubes. The concentrations of TNF-α, IL-1β, IL-6, IL-8, IL-10, IL-12, IL-17A, IFN-α, and IFN-γ were subsequently measured by Luminex technology and compared with corresponding values from a healthy reference group. P-values of ≤ 0.001 were considered statistically significant.

**Results:**

For patients with previous CA, decreased production of IFN-α, IFN-γ, and IL-12 was seen after stimulation of cells with LPS and Poly I:C (p ≤ 0.001). Moreover, cells from patients with previous cervical NSTI showed decreased production of IFN-α and IFN-γ after stimulation with LPS and Poly I:C (p ≤ 0.001). Nineteen out of 22 participants (86%) had oral pathologic conditions at the dental examination.

**Conclusion:**

The cytokine secretion for the LPS- and Poly I:C-stimulus suggests an impaired proinflammatory response within the innate immune response against bacterial and viral pathogens. A high prevalence of oral pathologic conditions was found at the clinical dental examinations. However, none of the patients experienced recurrence of CA or NSTI.

## Introduction

Odontogenic infections may develop from inconspicuous inflammatory conditions in the oral cavity including apical periodontitis, periodontitis and pericoronitis (Ogle, 2017). While many of these conditions remain localized, they can in some cases progress into serious systemic diseases. These infections range from abscesses and phlegmones to life-threatening infections such as cervical necrotizing soft tissue infections (NSTI) or secondary infections including infectious endocarditis, meningitis, liver-, lung-, spleen- and cerebral abscess (CA) (Parahitiyawa et al., 2009; Kilian et al., 2016; Gore, 2018; Peng et al., 2022; Bodilsen et al., 2024). Microorganisms often pass from the oral mucosa into the bloodstream, causing transient bacteremia. For reasons that remain unknown, and only in rare cases, they establish in extraoral tissues (Li et al., 2000; Parahitiyawa et al., 2009; Del Giudice et al., 2021). CA occurs when microorganisms encapsulate within the brain parenchyma, and the most common predisposing conditions are contiguous foci of infection, hematogenous spread of infections, head trauma, and neurosurgical procedures (Sonneville et al., 2017). CA usually presents with nonspecific symptoms such as headache, fever, nausea, neurological deficits, and in some cases seizures or signs of increased intracranial pressure. The clinical presentation of the disease is variable and depends on the location of the abscess, the number of abscesses, and their sizes (Helweg-Larsen et al., 2012; Brouwer and Van De Beek, 2017). The most common pathogens responsible for community-acquired CA are bacteria typically found in the oral cavity, including bacteria from the *Streptococcus anginosus* group (SAG), *Fusobacterium* spp., and *Aggregatibacter* spp (Bodilsen et al., 2024). Cervical NSTI presents as a rapidly progressive infection that results in widespread tissue destruction and is caused by direct spread of an ongoing infection along the subcutaneous fasciae which often involves oral microorganisms (Böttger et al., 2022). NSTI is characterized by pain, erythema, swelling, and is often accompanied by systemic complications such as fever, septic shock, and multi-organ failure (Hansen et al., 2023). The reason why few patients allow intracranial growth of oral microorganisms or the trigger for the aggressive transition from a local odontogenic infection into cervical NSTI is presently unknown. There is a high prevalence of asymptomatic oral pathological conditions in the general population (Eke et al., 2012; Tibúrcio-MaChado et al., 2021), and it is therefore striking that a larger proportion does not develop severe infections such as CA or NSTI. One possible explanation for an association could be a deviation in the immune response in patients who develop CA or cervical NSTI. The majority of individuals are able to prevent disseminated infections, but patients with immune system dysfunction have increased susceptibility to inflammation and establishment of infection (MaChado et al., 2004; Duffy et al., 2014). Recognition of bacterial molecular patterns via Toll-like receptors (TLRs) of cells belonging to the innate immune system is an early event in the induction of inflammation (Fitzgerald and Kagan, 2020), while T cells are activated further downstream. An altered or impaired immune response may be associated with a higher risk of CA or cervical NSTI. The aim of this study was to evaluate the dental characteristics and the stimulated immune response in previous CA and cervical NSTI patients, as we hypothesized that CA and cervical NSTI can be consequences of oral pathologic conditions in individuals with an altered immune response. We aimed to evaluate the stimulated immune response post-discharge, when the immune system was no longer influenced by an ongoing infection or treatment. The TruCulture technique was used where various TLR stimuli and T-cell-stimulating anti-CD3/-CD28 beads were performed. This technique has previously been used to characterize the immune responses in patients with Lyme neuroborreliosis (Ørbæk et al., 2021), patients undergoing hematopoietic cell transplantation (Gjærde et al., 2021) and in healthy individuals (Duffy et al., 2014). However, the TruCulture technique has not been used in evaluating previous CA or cervical NSTI patients.

## Methods and materials

### Setting and design

This observational study investigated the stimulated immune response and dental characteristics in previous CA or cervical NSTI patients. The study included a clinical dental examination, a digital panoramic radiograph (PR) and collection of blood samples for TruCulture analysis from participants that were previously treated for CA or cervical NSTI at Copenhagen University Hospital (CUH). Also, study variables from the medical records during hospitalization were utilized from previously published studies (Hansen et al., 2023; Jespersen et al., 2023). Participants were enrolled from Marts 2021 through September 2021, where they were examined at the Department of Odontology, University of Copenhagen. The stimulated immune response was compared with that of 32 anonymous healthy individuals. Permission to perform the study and to handle the data was granted by the Danish Data Protection Agency (record no. 3-3013-2579/1) and the Regional Scientific Ethical Committees of The Capital Region of Denmark (record no. H-20046532).

### Identification of participants, data source, and data extraction

CA patients were identified through the Danish Study Group of Infections of the Brain (DASGIB) database, and cervical NSTI patients were identified through the Improving Outcome of Necrotizing Fasciitis (INFECT) database. Both databases are prospective observational databases containing predefined study variables for CA and NSTI patients during hospitalization. The DASGIB database includes patients hospitalized between January 1, 2015, and December 31, 2020, while the INFECT database includes NSTI patients hospitalized between 2013 and 2017. Both databases follow strict, prospectively defined inclusion protocols to ensure diagnostic accuracy. The databases have been described in detail previously (Madsen et al., 2019; Bodilsen et al., 2021).

### Inclusion and exclusion criteria

**Figure 1:**
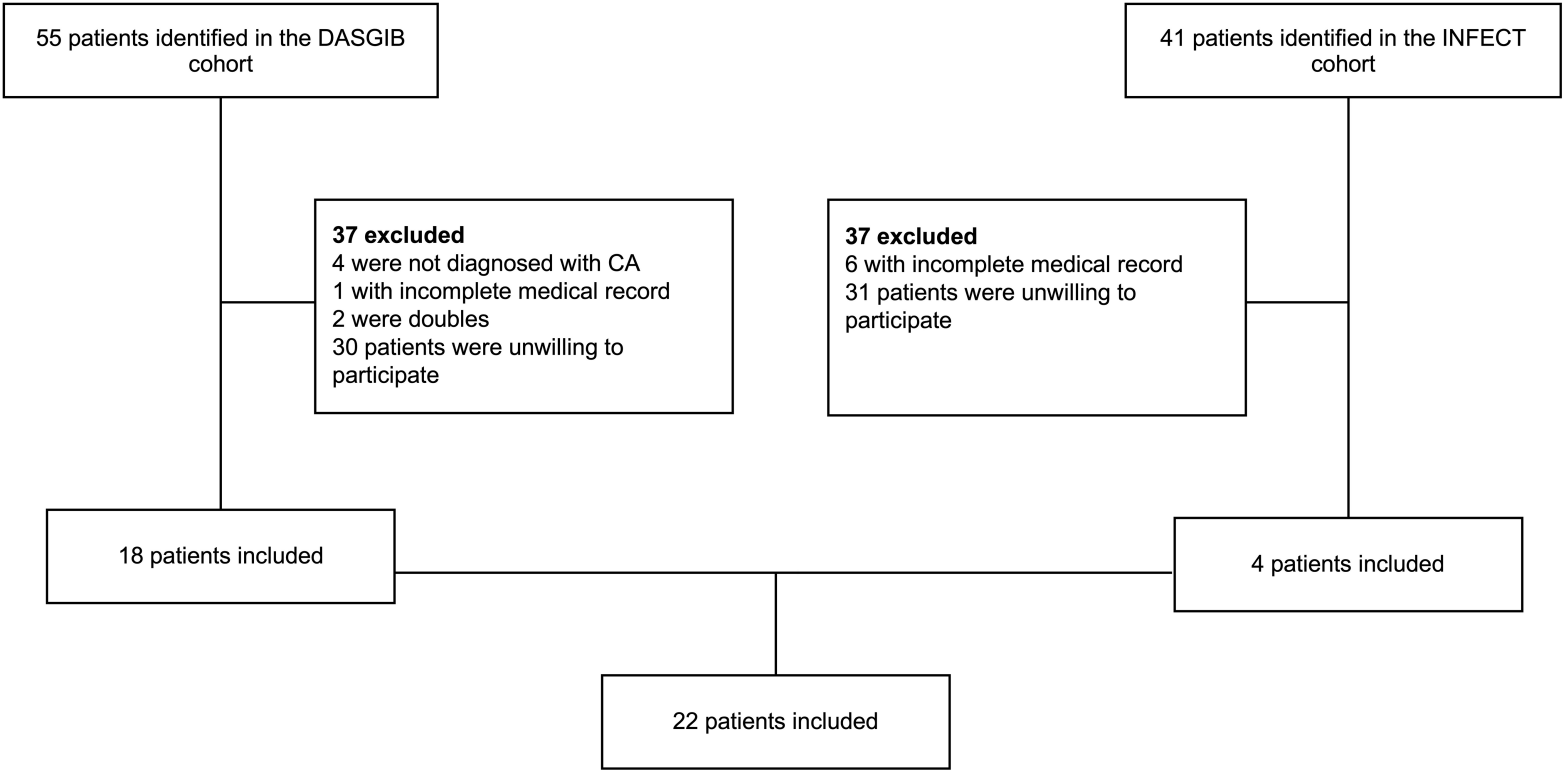
Flow of patients. All patients from the DASGIB and INFECT cohort were screened for eligibility. Patients were excluded if they did not meet the inclusion criteria. In total, 22 patients were included.

Participants were included in this study if they fulfilled the following criteria: 1) Had previously been diagnosed with CA or cervical NSTI with a minimum of 6 months recovery, 2) Were ≥ 18 years of age, and 3) Had given informed consent to participate. The flow of participants is illustrated in **Figure 1**. All eligible patients were included and no certain groups of previous CA or cervical NSTI patients were selected.

### Participant recruitment

All Danish residents have a unique civil registration number which is linked to a personal digital mailbox system (e-Boks). Eligible patients in the DASGIB and INFECT databases had their civil registration numbers identified and were contacted through e-Boks in January 2021. Patients who did not respond on e-Boks requests were contacted by phone if telephone numbers could be retrieved from their medical records.

### Clinical and radiographic examination

Participants had recovered from their CA or cervical NSTI infection before the clinical examination. The clinical examinations were performed in cooperation by two last-year dental students, Frederik Viktor Bang Jespersen (FVBJ) and Signe Undall-Behrend Hansen (SUBH). Study variables were collected including gender, age, general diseases, medication, allergies, smoking habits, and alcohol consumption. Full-mouth clinical examination included systematic registration of decayed, missed, filled, teeth (DMFT), retained teeth, and periodontal status including probing pocket depth (PPD), clinical attachment loss (CAL), and bleeding on probing (BOP) at six sites per tooth. Gingivitis and periodontitis were registered, if the criteria were fulfilled based on the classification from the workshop arranged by the American Academy of Periodontology and the European Federation of Periodontology in 2017 (Chapple et al., 2018; Papapanou et al., 2018). PRs were taken during the clinical examination at the Department of Odontology, University of Copenhagen. All PRs were evaluated by the two observers FVBJ and MM on six radiographic variables: The presence of endodontically treated teeth, retained teeth, signs of oral pathologic conditions like deep carious lesions, periapical radiolucencies, severe marginal bone loss, and pericoronal radiolucencies. The observers were considered calibrated based on a previous study evaluating the same parameters on PRs, where the level of agreement on the presence of oral pathologic conditions was interpreted as substantial to almost perfect among the observers (Jespersen et al., 2023).

### Blood samples and TruCulture analysis

A custom-designed TruCulture panel has been developed at CUH to comprehensively assess the induced immune response. The TruCulture panel used in this study was composed of four different immune cell stimulations and was constructed to replicate the immune response to different viral and microbial stimulations, hereby evaluating the function of extracellular and intracellular immunological signaling pathways. The TruCulture panel consisted of the following immune cell stimuli: *E. coli* LPS (a TLR4 agonist), R848 (resiquimod, a dual TLR8 and TLR7 synthetic agonist), Poly I:C (a TLR3 agonist), and anti-CD3/-CD28 that activates T-cell receptors (TCR) (Duffy et al., 2014). Additionally, a null-tube without any stimulating agent was included.

Participants had 9 mL of whole blood sampled in lithium heparin tubes for TruCulture analysis. Blood samples were collected by a trained nurse. The handling of the blood samples were identical to the methodology used in a previous study (Gjærde et al., 2021): Within one hour from blood sampling, 1 mL of whole blood was distributed into five prewarmed TruCulture (Myriad RBM) tubes. The tubes were inserted into a digital dry block heater (WWR International A/S), maintained at 37°C for 22 hours according to manufacturer’s protocol. The tubes were opened, and a valve was inserted to separate sedimented cells from the supernatant and to stop the stimulation reaction. The liquid supernatants were aliquoted and immediately frozen at -20°C and transferred to -80°C after 1–7 days until use. The secretion of nine different cytokines - TNF-α, IL-1β, IL-6, IL-8, IL-10, IL-12, IL-17A, IFN-α, and IFN-γ - were measured by an Luminex bead set (LX200, R&D Systems, BIO-Teche LTD) according to the manufacturer’s protocol. Cytokine secretion in tubes containing patient cells were compared to those obtained in tubes containing cells from a healthy reference group the consisted of 32 anonymous healthy individuals aged between 18 and 67 years. Cytokine secretion was available for all 32 individuals, except IFN-α that was limited to 16 reference individuals for the null-tube and the LPS-, R848- and Poly I:C-stimulus. For the anti-CD3/-CD28-stimulus, all nine cytokines were limited to 18 reference individuals.

### Statistical analyses

**Figure 2:**
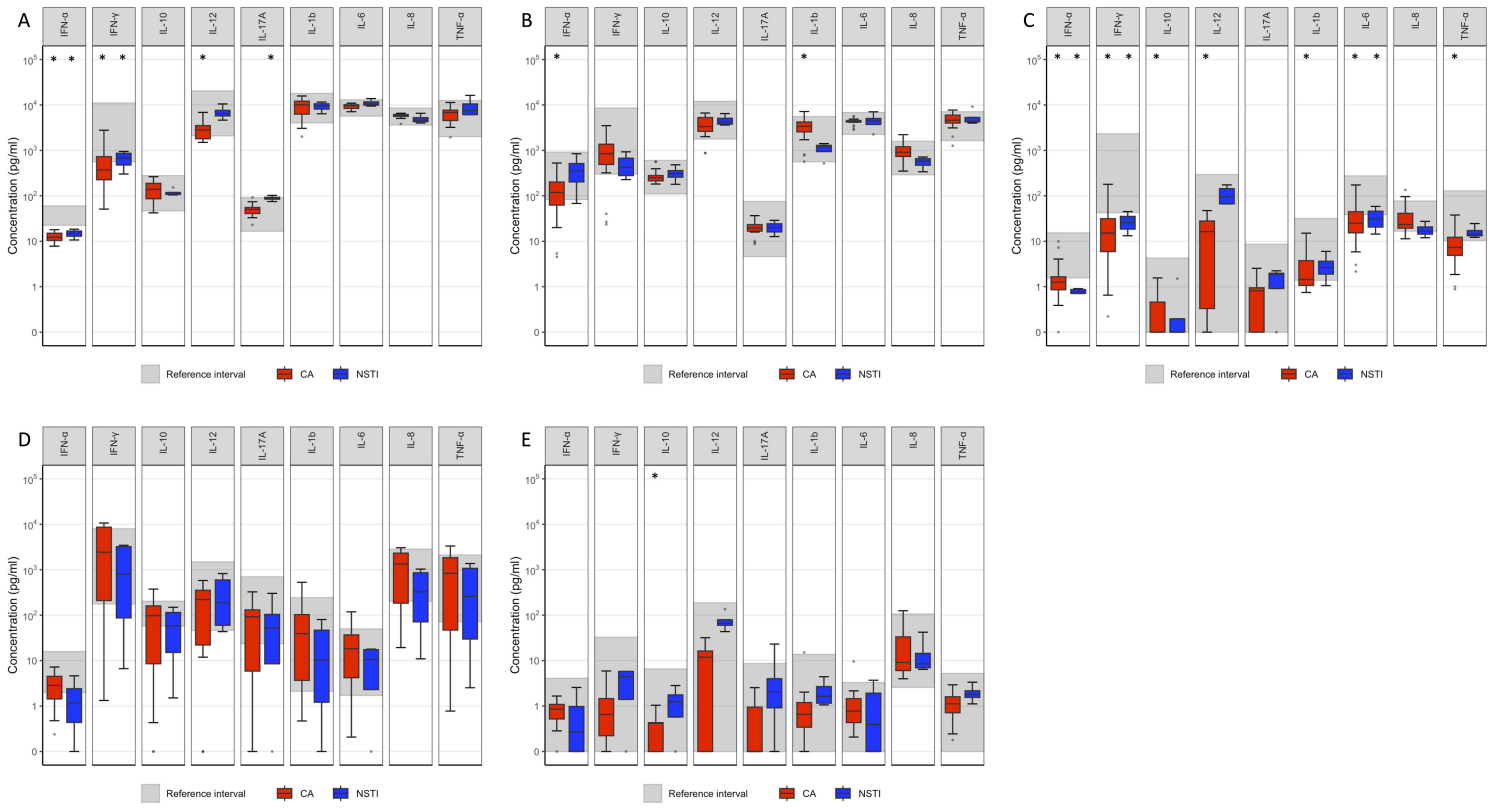
Cytokine response by TruCulture test. The grey area represents the reference interval. Comparison between cytokine concentration in blood from patients with previous CA and cervical NSTI with the reference interval, respectively. *p ≤ 0.001. **(A)** LPS **(B)** R848 **(C)** Poly I:C **(D)** anti CD3/-CD28 **(E)** Null. All concentrations have been added a value of 0.1, to accommodate concentrations of 0. Data points beyond the end of whiskers are plotted individually.

All statistical analyses were performed using SPSS Statistics version 28.0 (IBM Corporation, Armonk, NY). Data distribution was assessed using visual inspection and the Shapiro–Wilk test. For comparisons between CA patients and the reference group, independent samples t-tests were used for normally distributed data, while the Mann–Whitney U test was applied for non-normally distributed data. For comparisons involving cervical NSTI patients and the reference group, the Mann–Whitney U test was used due to the small sample size. A Bonferroni correction was applied to adjust for multiple comparisons, and the significance level was set at p ≤ 0.001 (two-sided, exact). **Figure 2** was generated using the same R script as in a previous study (Gjærde et al., 2021).

## Results

### Characteristics at the examination

A total of 22 participants was included in this study (**Figure 1**). Background data on the study population are detailed in **Table 1**. Eighteen participants (82%) were previous CA patients, and 16 (73%) were males. The mean period from the day of discharge from CUH to the clinical examination and blood sample was 3 years (1073 days), with a minimum of two years since discharge. None of the participants had been diagnosed with recurrence of CA or cervical NSTI since their hospitalization. During the clinical examination, four participants (18%) reported having type 2 diabetes (T2D), but details on the glycemic control in these participants were not available. Other non-communicable diseases such as hypertension and hypercholesterolemia were present in 9 (41%) and 5 (22%) patients respectively. One patient had been diagnosed with myelomatosis and therefore received chemotherapy including the immunosuppressive drugs lenalidomide and ixazomib. This patient was the only one receiving immunosuppressive drugs. None of the participants reported drinking ≥14 units (equivalent to 168 grams) of alcohol per week, nor did any report any drug abuse. Four patients were smokers and reported to smoke ≥10 cigarettes a day. Twelve CA patients were characterized as having a CA of odontogenic origin during hospitalization using criteria from a previous study (Jespersen et al., 2023). In the remaining six CA patients, three patients had suspected origin of CA due to ear infection, two due to sinusitis, and one with unknown origin. Two cervical NSTI patients had a suspected odontogenic origin using criteria from a previous study during hospitalization (Hansen et al., 2023) and two had a peritonsillar abscess as suspected origin of infection.

**Table 1:** Patient characteristics.

Characteristics	Total, n (%)
Patients	22 (100)
Previous CA	18 (82)
Age, years, mean ± SD	62 ± 11
Gender, male	16 (73)
Smoking	
None	18 (82)
≥ 10 cigarettes/day	4 (18)
Alcohol consumption	
None	7 (32)
≥ 1 ≤ 7 units/week	12 (55)
≥ 8 ≤ 14 units/week	3 (14)
Polypharmacy^A^	6 (27)
Comorbidities and immunocompromising factors^B^	
None	7 (32)
Diabetes type two	4 (20)
Hypertension	8 (41)
Hypercholesterolemia	5 (22)
Cancer (Myelomatosis)	1 (5)

CA, Cerebral abscess; SD, Standard deviation.

^A^Defined as regular use of at least five different types of medication.

^B^4 patients had more than one comorbidity.

### Clinical dental examination

Dental characteristics are detailed in **Table 2** and the oral pathologic conditions, as assessed clinically and radiographically, in **Table 3**. The oral and radiographic examinations revealed oral pathologic conditions in 19/22 patients (86%). The clinical examinations revealed gingivitis in two patients, periodontitis in 10 patients, and pericoronitis in two patients. Four participants were diagnosed with stage II periodontitis and six were diagnosed with stage III periodontitis. For the PR evaluation, the two examiners were in complete agreement in 149/154 observations (97%) and reached consensus on the remaining five observations. The examiners agreed on the presence of radiographic pathologic conditions in 19/22 patients (86%). The examination revealed apical radiolucencies in 13 patients, severe marginal bone loss in 9 patients, and pericoronal radiolucencies in three patients. Multiple oral pathologic conditions were present in 10 patients. One patient was registered with severe bone loss on the PR but had no clinical signs of periodontitis. Also, one patient was registered with pericoronal radiolucency, but did now show clinical signs of pericoronitis. Both patients were registered without oral pathologic conditions. In the 12 odontogenic CA patients, apical periodontitis was registered in six patients, periodontitis in seven patients, pericoronitis in two patients and gingivitis in one patient. For the six non-odontogenic CA patients, apical periodontitis was registered in four patients, periodontitis in three patients and gingivitis in one patient. In the two odontogenic NSTI patients, apical periodontitis was registered in one patient and for the two non-odontogenic NSTI patients apical periodontitis was registered in both. Dental caries was registered in 13 patients with superficial carious lesions in three CA patients and two NSTI patient, media lesions in 8 CA patients and two NSTI patients and profund lesions extending to the pulp chamber in one NSTI patient. Multiple carious lesions were present in four patients.

**Table 2:** Dental characteristics.

Dental characteristics	Mean ± SD
Number of teeth	25 ± 4.9
DMFT	15.5 ± 5.5
PPD, mm	2.4 ± 0.5
BOP, %	8 ± 7.9
CAL, mm	2.6 ± 1.5

DMFT, Number of decayed, missed and filled teeth; PD, Pocket depth; BOP, Bleeding on probing; CAL, Clinical attachment loss.

**Table 3:** Oral pathologic conditions in previous CA and cervical NSTI patients.

Oral pathology	n (%)
**Clinical oral pathology**	13 (60)^a^
Gingivitis	2
Periodontitis	10
Stage II	4
Stage III	6
Pericoronitis	2
Dental caries	13
Superficial	5
Media	10
Profund	1
**Radiographic oral pathology**	19 (86)^b^
Apical radiolucency	13
Severe marginal bone loss	9
Pericoronal radiolucency	3

CA, cerebral abscess; NSTI, necrotizing soft tissue infection.

^a^1 patient had more than one clinical oral pathologic condition.

^b^10 patients had more than one radiographic oral pathologic condition.

### TruCulture analysis

**Figure 3:**
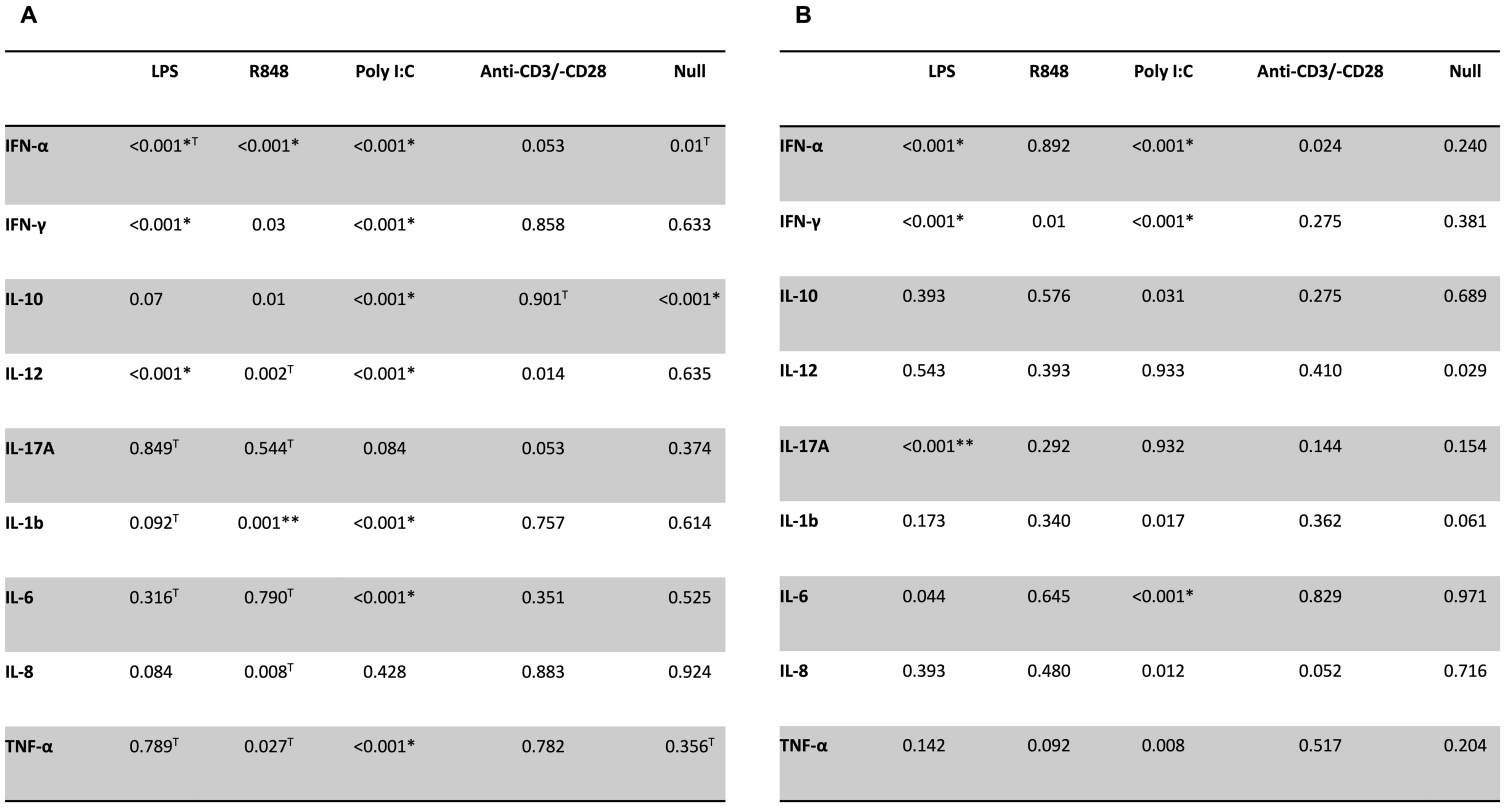
Comparison of P-values by TruCulture test. P-values for differences in cytokine concentrations between reference group and patients with previous CA **(A)** or cervical NSTI **(B)**. *Lower than reference group, **Higher than reference group. ^T^Tested using independent samples t-tests.

The results of the TruCulture test are presented in **Figures 2** and **3**. For previous CA patients, the unstimulated secretion of cytokines for the null-tube was comparable to that of the reference group, except for the secretion of IL-10 which was significantly decreased (p ≤ 0.001). However, the previous CA patients’ cells showed decreased LPS-induced secretion of IFN-α (p ≤ 0.001), IFN-γ (p ≤ 0.001) and IL-12 (p ≤ 0.001) and decreased R848-induced secretion of IFN-α (p ≤ 0.001), while the secretion of IL-1β was increased after stimulation with R848 (p ≤ 0.001). Following stimulation with poly I:C, cells from these patients showed decreased secretion of all cytokines measured (p ≤ 0.001), except for IL-17A and IL-8. No significant difference in cytokine secretion was observed after stimulation with anti-CD3/-CD28.

For previous NSTI patients, the unstimulated secretion of cytokines for the null-tube showed no significant difference from those of the reference group for any of the cytokines measured, but stimulation with LPS induced a decreased secretion of IFN-α (p ≤ 0.001) and IFN-γ (p ≤ 0.001), and an increased secretion of IL-17A (p ≤ 0.001). Accordingly, the previous NSTI patients’ cells showed lower secretion of IFN-α, IFN-γ, and IL-6 (p ≤ 0.001) than cells from the reference group after stimulation with poly I:C. No significant differences were observed in cultures stimulated with R848 or anti-CD3/-CD28 beads.

## Discussion

This is the first study to report an oral clinical examination and simultaneously investigate the stimulated immune response in patients who previously suffered from CA or cervical NSTI. The hypothesis that these patients have an impaired innate immune response when compared to a reference group was corroborated, while no such impairment of the adaptive immune system was observed. Almost all patients had oral pathologic conditions at the oral examination, with periodontitis and apical periodontitis as the dominating conditions. The DMFT score was similar to what is generally expected for the same age group in Denmark (Kongstad et al., 2013). The oral examination indicate that apical periodontitis was prevalent in both odontogenic and non-odontogenic CA and NSTI cases, whereas periodontitis and pericoronitis were observed exclusively in CA patients. The prevalence of periodontitis and apical periodontitis in this study aligns with estimates from the general population, where these conditions are believed to affect approximately 50% of individuals (Eke et al., 2012; Tibúrcio-MaChado et al., 2021).

Cells from previous CA patients showed decreased secretion of IFN-α, IFN-γ, and IL-12 after stimulation with the TLR4 agonist *E. coli* LPS and the TLR3 agonist poly I:C. Moreover, cells from previous cervical NSTI patients also showed reduced secretion of IFN-α and IFN-γ after these stimuli, indicating an impaired innate immune response towards gram-negative bacteria and viruses in both patient groups.

The cytokine secretion induced by the LPS stimulus is of particular interest, as CA and cervical NSTI are infectious diseases caused primarily by bacteria. IFN-γ plays a key role in regulating the innate and adaptive immune system (Shtrichman and Samuel, 2001) and the expression of the cytokine is regulated through a series of interactions involving macrophages and dendritic cells, as well as T cells, natural killer cells (Varma et al., 2002) and microglia cells in the brain (Luckheeram et al., 2012). IFN-γ serves to enhance the antimicrobial immune response by stimulating multiple macrophage functions, promoting the secretion of opsonizing immunoglobulin by B cells, promoting T-helper 1 (Th1) differentiation, maturation of cytotoxic T cells and activating neutrophils (Varma et al., 2002). It has previously been shown that IFN-γ-receptor deficient mice have increased susceptibility to dissemination of bacterial pathogens compared to wild-type mice (Kamijo et al., 1993), as well as an increased risk of bacterial infections due to reduced macrophage activation (Tau, 1999). Oral streptococci closely interact with macrophages at the oral mucosal surface (Matsushima et al., 2017), and impaired macrophage activity may increase the risk of disseminating pathogens. In a previous study, oral infections with *Fusobacterium nucleatum* in mice induced significantly higher levels of IFN-γ. IFN-γ is therefore believed to play an important role in the response against this microorganism in the oral cavity (Johnson et al., 2018). Interestingly, *F. nucleatum* is often isolated from CA lesions (Kommedal et al., 2014), cervical NSTI (Böttger et al., 2022), and from infectious oral conditions (Jespersen et al., 2023). A previous study using TruCulture has also suggested, that a low LPS-stimulated IFN-γ is associated with a higher risk of bacterial infections (Gjærde et al., 2021). Cell cultures from previous CA patients also contained low secretions of IL-12 after stimulation with LPS. IL-12 is primarily secreted by activated macrophages, triggering the production of IFN-γ (Tau, 1999). Thus, both IL-12 and IFN-γ play critical roles as cytokines that initiate the downstream signaling cascade for the development of Th1 cells which makes these cytokines a link between natural and adaptive immunity (Romani et al., 1997) and essential for overcoming a range of bacterial infections (Schmidt et al., 2016). Additionally, low secretion of IFN-α were observed in cell cultures from both patient groups. IFN-α is known as a potent antiviral protein; however, recent studies have also demonstrated its role in the immune response to bacterial pathogens (Bogdan, 2000). The production of IFN-α is upregulated when macrophages are exposed to microbial pathogens and microbial products including LPS (Bogdan, 2000). IFN-α has also been shown to stimulate the production of IL-12 and IFN-γ (Bogdan, 2000; Iellem et al., 2000). Thus, IFN-γ, IL-12, and IFN-α are all important in initiating an effective immune response against bacterial infections (Bogdan, 2000; Schroder et al., 2004).

The poly I:C stimulus mimics a viral infection, and the production of IFN-α and IFN-γ was significantly decreased in both patient groups for this stimulus. This finding may translate into an increased risk or altered response to viral infections. The normal immune response to anti-CD3/-CD28 stimulus. This does not exclude a relative B-cell deficiency and thereby decreased production of antibodies. The cytokine secretions measured for the null-stimulus reflects the resting state of the immune system, and neither patients with previous CA nor patients with previous cervical NSTI showed abnormal baseline cytokine responses, except for a decreased production of IL-10 in patients with previous CA. This finding indicates a relatively normal baseline level of cytokines before stimulation in previous CA and cervical NSTI patients.

Oral diseases such as periodontitis and apical periodontitis are chronic inflammatory diseases that are associated with systemic inflammation (D’Aiuto et al., 2018; Bakhsh et al., 2022). The link between periodontitis and systemic inflammation has gained much attention in recent years and the heightened systemic inflammation is likely caused by a spillover of inflammatory mediators from the periodontal tissue to the bloodstream and hematogenous dissemination of periodontal bacteria (Hajishengallis and Chavakis, 2021). Oral diseases increase the systematic inflammatory burden, and a recent study found that periodontal treatment successfully reduced systemic inflammatory markers (D’Aiuto et al., 2018). Also, treatment of periodontitis and apical periodontitis are associated with improvements in circulating levels of C-reactive protein (Botelho et al., 2022). These findings are particularly interesting from a CA perspective, as numerous studies have shown, that systemic inflammation reduces the integrity of the blood-brain-barrier (BBB) and is thereby associated with heightened BBB permeability (Galea, 2021). Bacteria may cross the BBB by transcellular transportation, paracellular transportation, or via infected leukocytes, and host inflammatory factors disrupting the BBB may occur during bacterial CNS invasion (Dando et al., 2014). Predisposing factors for the development of CA include infections, congenital heart diseases, and immunocompromising conditions (Bodilsen et al., 2019) however, other factors increasing the risk of CA may include genetic predispositions, age, sex, infections, and systemic inflammation that are all known to increase the BBB permeability (Hajishengallis and Chavakis, 2021). It is well known that systemic inflammation, including diabetes, increases the risk of NSTI (Cheng et al., 2015). Consequently, oral diseases may also increase the risk for cervical NSTI by contributing to systemic inflammation.

None of the patients experienced recurrence of CA or NSTI or developed other severe infections after their hospitalization despite having oral pathologic conditions after the course of the disease. This corresponds well with the existing literature, reporting very rare cases of recurrent CA or NSTI (Kuzdan et al., 2011; Helweg-Larsen et al., 2012). The altered cytokine response suggests a susceptibility to both bacterial and viral infections. One possible explanation for why patients develop CA or cervical NSTI is that concurrent infections may be required to trigger disease development. This could explain their rare recurrence in patients with altered cytokine profiles. It is well established that there is an interplay between viral and bacterial infections in various infectious diseases including otitis media, sinusitis, and respiratory tract infections (Bakaletz, 2009; Ferreira et al., 2011; Manna et al., 2020). Viral RNA has also been isolated from oral pathologic conditions including periapical abscesses (Ferreira et al., 2011) and it is well known, that certain viruses exert immune suppressive functions, which may lead to increased risk of bacterial super infections (Beadling and Slifka, 2004). Viral infections can enhance bacterial adherence and infiltration, enabling bacteria that are harmless locally to spread systemically (Beadling and Slifka, 2004; Eke et al., 2012; Manna et al., 2020; Feldman and Anderson, 2021). The need for a concurrent presence of both viral and bacterial infections may be one of the potential mechanisms behind the rare recurrence of CA and cervical NSTI patients. However, compensatory adaptive immune responses may also contribute to preventing recurrence of these infections. However, the pathogenesis is likely multifactorial, involving environmental, genetic, anatomic, microbial, immunological, and possibly concurrent infection related factors. CA and cervical NSTI may be consequences of oral pathologic conditions in individuals with an impaired immune response, as the production of IFN-γ, IL-12, and IFN-α may play a role in the pathogenesis of CA and cervical NSTI. However, the low recurrence rate combined with oral pathologic conditions suggests that additional factors are crucial in the disease development.

## Strength and limitations

The main strength of the present study is the well-defined cohorts of patients with previous CA or cervical NSTI. The data from the clinical and radiographic examination were prospectively collected, and data from the hospitalization were collected in a predefined registration form. The TruCulture method and the use of Luminex are reliable methods for cytokine analysis, and the use of Mann-Whitney U and Bonferroni correction ensures a robust statistical analysis. Another strength is that the cytokine concentrations measured for the null-stimulus act as a negative control, and most of them were comparable with the reference group. Moreover, a previous study using the same anonymous reference group have been able to identify differences between the healthy reference group and an experimental group (Ørbæk et al., 2021).

However, there are also limitations in this study. The small sample size limits the ability to detect subtle immune alterations or subgroup variations. A larger cohort could provide deeper insights into functional immune responses and allow for a more detailed analysis of potential differences based on infection type, severity, or treatment history. The immune cell stimuli were compared with an anonymous in-house reference interval rather than a control group matched for age, gender, leukocyte count, or oral pathologic conditions, limiting the comparability between patient and reference groups. Another limitation is the study design, which only captures immune responses at a single time point post-recovery. Longitudinal data would be needed to assess whether the observed immune alterations persist over time. Furthermore, the lack of a matched control group with dental data makes it difficult to determine the precise relationship between specific oral pathologic conditions and immune alterations in this cohort. Finally, there may be selection bias, as all patients had recovered for at least two years. Individuals with more severe immune dysregulation or recurrent infections may not be represented, potentially underestimating immune alterations in post-recovery of CA and NSTI patients.

## Conclusion

Impaired proinflammatory cytokine response to the LPS and poly I:C suggest an altered innate immune response against bacterial and viral pathogens among patients with previous CA or cervical NSTI, which may allow bacterial pathogens from inflammatory conditions in the oral cavity to evade the innate immune system. The normal cytokine response to stimulation with anti-CD3/-CD28 are indicative of a normal T-cell response in these patients. Almost all patients had oral pathologic conditions at the oral examination, with periodontitis and apical periodontitis as the dominating conditions. This study was explorative, and further studies are needed to fully understand the underlying mechanisms of the present findings.

## Data Availability

Due to General Data Protection Regulation constraints, the raw data of stimulated cytokine measurements cannot be disclosed. However, summary statistics, including medians, means, standard deviations, and interquartile ranges, will be made available upon request. Additionally, data from the clinical dental examinations can also be provided upon request. Requests to access the datasets should be directed to frederik.vb.jespersen@sund.ku.dk.
